# Comparison Between Direct, Virtual Aided by Clinician and Artificial Intelligence Bonding Techniques in Orthodontics

**DOI:** 10.1111/ocr.70032

**Published:** 2025-10-13

**Authors:** Tarek ElShebiny, Johana Cortés‐Mercado, Neda Stefanovic, Juan Martín Palomo

**Affiliations:** ^1^ Department of Orthodontics Case Western Reserve University Cleveland Ohio USA; ^2^ Department of Orthodontics University of Belgrade Belgrade Serbia

## Abstract

**Objectives:**

The purpose of this study was to determine if there are any clinically significant differences between direct, virtual indirect, and artificial intelligence (AI) bonding techniques.

**Materials and Methods:**

This in vivo study analysed 840 teeth selected from 14 patients undergoing orthodontic treatment with full fixed appliances. Anatomical superimpositions were performed, and data were collected as both numerical values and colour‐coded deviation maps to assess the differences between direct, AI, and virtual indirect bonding techniques.

**Results:**

The intraclass correlation coefficient test showed good correlation (0.894). The Kruskal–Wallis comparison showed a statistically significant difference when comparing direct to virtual indirect and direct to AI. Descriptive statistics showed 4 values with clinically significant differences when comparing direct to virtual indirect. Descriptive statistics showed 3 values with clinically significant differences when comparing direct to AI. Root mean square (RMS) discrepancies exceeding 0.50 mm were found in four tooth types (AI vs. Direct) and three (Clinician vs. Direct).

**Conclusions:**

We found statistically and clinically significant differences between AI and virtual indirect when compared to direct bonding. With our data, we could infer that if we compare AI versus virtual indirect, there might not be any clinically significant differences since the differences between them fall below 0.25 mm.

## 1 Introduction

Bracket positioning is one of the most critical stages of orthodontic treatment [1]. That is why the advantages and disadvantages of direct and indirect bonding techniques have been discussed constantly in the orthodontic world [2, 3, 4, 5, 6, 7, 8, 9]. The most commonly used technique, direct bonding, undoubtedly carries some disadvantages like long bonding time, need for wire bending in later stages of treatment, bracket repositioning and bracket positioning inaccuracies [1]. Direct bonding is believed to take more clinical chair time, therefore adding more stress to the orthodontist [3, 4, 5]. Even though indirect bonding has been advocated for many years to improve efficiency and outcome of treatment, this technique is still only being used by a minority of orthodontists [7, 8, 9, 10]. It is important to mention that indirect bonding is more technique sensitive and requires more laboratory procedures and time, besides chair time [8, 9, 10, 11]. Digital developments like intraoral scanning and 3D printing are now very advanced and more accessible to orthodontists [2, 12]. Orthodontists are well aware of the quick and precise options for capturing the dentition and the occlusion using intraoral scanners. The accuracy of this technology is very well documented and acceptable for daily clinical orthodontics [2].

These digital advances made the development of the virtual indirect bonding technique possible [6, 7, 8]. Recently, a variety of software programs started offering virtual bracket placement with the aid of artificial intelligence (AI). Digital orthodontic bonding, with the integration of AI, could enhance the precision and efficiency of the bonding procedure, resulting in improved treatment outcomes and patient satisfaction. Prior AI‐based work in orthodontics has focused on automating various aspects of diagnosis and treatment planning, including cephalometric landmark detection, growth prediction, and anatomical segmentation. Software solutions now use machine learning algorithms trained on large clinician‐labelled datasets to predict optimal bracket positions on virtual dental models. However, the clinical applicability and accuracy of AI‐assisted virtual bonding under real in vivo conditions remain underexplored, particularly in direct comparison with conventional clinician‐guided virtual indirect bonding techniques [13, 14, 15, 16]. The purpose of this study was to determine whether there were any significant differences between direct bonding, virtual indirect bonding, and AI‐aided indirect bonding techniques.

## 2 Materials and Methods

The sample of this study consisted of 14 patients who started orthodontic treatment with full fixed appliances at a private practice. All subjects were in permanent dentition. Patients with abnormal tooth morphology, restoration(s) that altered the natural morphological integrity of the crown, poor oral hygiene, craniofacial anomalies, or syndromes were excluded. The sample size calculation was based on detecting a 0.5 mm clinically relevant RMS difference (American Board of Orthodontics (ABO) threshold) with an SD of 0.22 mm, consistent with a prior indirect bonding study [7]. The sample size calculation showed that at least 14 dental arches would be needed for each group to detect a minimum root mean square (RMS) clinical difference of 0.50 mm and a standard deviation of 0.22 mm with a power of 80% and a significance level of 0.05.

In our study, we used the iTero Element 2 IO scanner (Align Technologies, San Jose, Calif) and 3M Victory Low Profile MBT brackets, slot 0.022 (3M Unitek, Monrovia, Calif), both in vivo and virtually.

A digital intraoral scan (IO scan) was taken at the initial records appointment for each subject. A second digital IO scan was taken after direct bonding. The initial scan taken prior to direct bonding was submitted to Dibs AI software (American Fork, UT) for digital bracket placement. The DIBS AI software uses a proprietary machine learning algorithm trained on thousands of clinician‐verified bracket placements to predict optimal bracket positioning relative to the clinical crown centre. Direct bonding was performed manually using standard visual placement without calibrated jigs or altimeters, replicating routine clinical practice. Each initial IO scan was submitted twice: the first time to evaluate the AI‐aided bracket placement without clinician's modifications, and the second time for a virtual setup with clinician's modifications. Three bracket placement options were assessed and compared: AI‐aided virtual bracket placement, clinician‐aided virtual bracket placement, and direct bonding in vivo.

**FIGURE 1:**
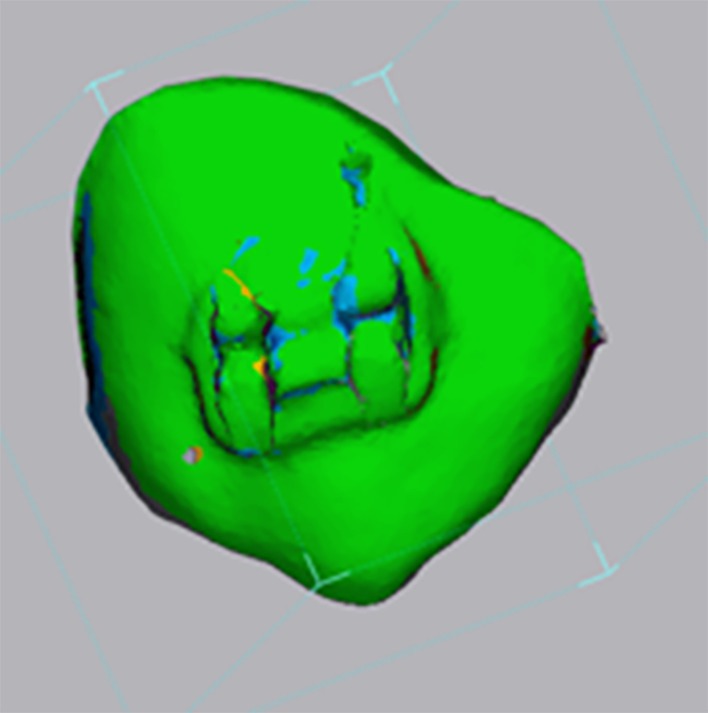
Superimposition and colour mapping tool example using Geomagic Control X software between UL3 Direct Bonding and UL3 AI.

**FIGURE 2:**
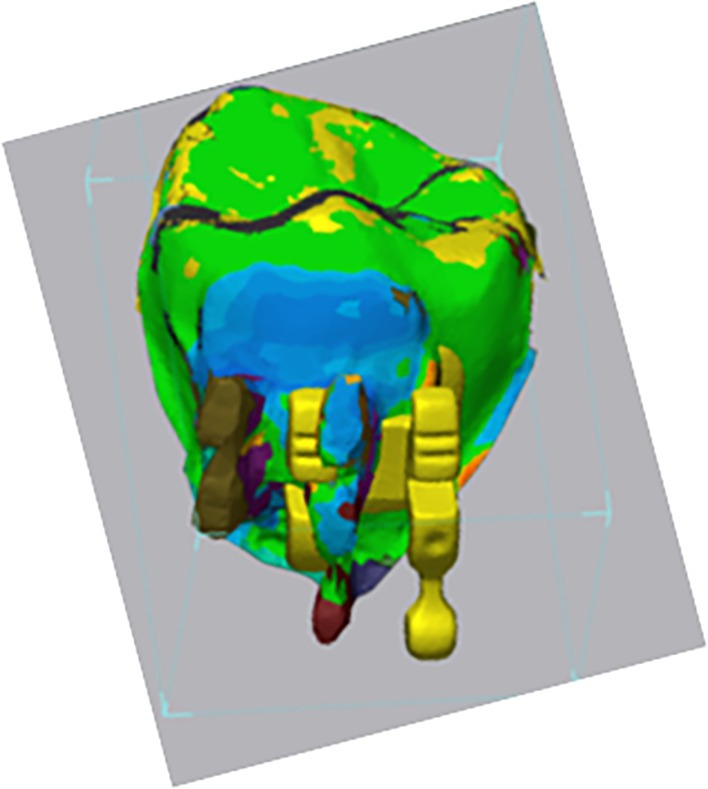
Superimposition and colour mapping tool example using Geomagic Control X software between Premolar direct bonding and virtual indirect bonding.

After obtaining stereolitography (.stl), files of the three bracket placement modalities arches were segmented into individual tooth units using the sculpting tool from Dolphin Imaging Software. Geomagic Control X software (3D mesh processing software) was then utilised to superimpose individual segmentations to assess bracket placement differences using the direct bonding IO scan as the reference. Superimpositions were performed on the occlusal surfaces of the segmented teeth, and data were collected as both numerical values and colour‐coded deviation maps. (Figures 1 and 2) All the statistical analyses were performed using SPSS 22.0 software (IBM, Armonk, NY, USA). Descriptive statistics were computed, and statistical significance was set at *p* < 0.05. The Shapiro–Wilk test showed that the data were not normally distributed. Therefore, nonparametric testing was performed to evaluate our data. The Kruskal–Wallis test was conducted to assess statistically significant differences between bracket positioning options. (Tables 1 and 2).

**TABLE 1:** Descriptive statistics Kruskal–Wallis Group A (Direct Bonding vs. AI).

		Sum of squares	df	Mean square	*F*	Sig.
RMS	Between groups	1.46	9	0.16	13.10	< 0.001
	Within groups	3.35	270	0.01		
	Total	4.82	279			
Max.	Between groups	0.73	9	0.08	11.11	< 0.001
	Within groups	1.98	270	0.01		
	Total	2.72	279			
Min.	Between groups	0.73	9	0.08	11.11	< 0.001
	Within groups	1.98	270	0.01		
	Total	2.72	279			

**TABLE 2:** Descriptive statistics Kruskal–Wallis Group B (Direct Bonding vs. Virtual Clinician).

		Sum of squares	df	Mean square	*F*	Sig.
RMS	Between groups	0.97	9	0.11	10.54	< 0.001
	Within groups	2.75	270	0.01		
	Total	3.72	279			
Max.	Between groups	0.56	9	0.06	5.91	< 0.001
	Within groups	2.82	270	0.01		
	Total	3.37	279			
Min.	Between groups	0.59	9	0.07	6.28	< 0.001
	Within groups	2.83	270	0.01		
	Total	3.43	279			

## 3 Results

A total of 840 teeth (central incisors to second premolars) were evaluated. The intraclass correlation coefficient test showed good correlation (0.894). For this study, we were studying (A) the differences in bracket positioning between the direct bonding (DB) and the virtual bonding done by AI. (B) Differences in bracket positioning between the DB method and the indirect virtual bonding done by the clinician (Clin).

### 3.1 Group A: Direct Bonding Versus AI

The Kruskal–Wallis comparison test showed a statistically significant difference between all the values: RMS, minimum, and maximum values, with a *p*‐value of < 0.05 (Table 1, Supplement S1).

Descriptive statistics in Table 3 show 4 values with clinically significant differences in this group (> 0.50 mm): mandibular lateral incisors (0.60 mm), maxillary lateral incisors (0.56 mm), maxillary second premolars (0.54 mm), and maxillary first premolars (0.53 mm).

**TABLE 3:** Descriptive statistics Direct Bonding versus AI.

Teeth	RMS	Max	Min (−)
U1	0.45 ± 0.11	0.82 ± 0.05	0.82 ± 0.05
U2	**0.56 ± 0.15**	0.82 ± 0.09	0.82 ± 0.09
U3	0.40 ± 0.06	0.73 ± 0.07	0.73 ± 0.07
U4	**0.53 ± 0.14**	0.83 ± 0.05	0.83 ± 0.05
U5	**0.54 ± 0.13**	0.84 ± 0.10	0.84 ± 0.10
L1	0.43 ± 0.10	0.78 ± 0.13	0.78 ± 0.13
L2	**0.60 ± 0.15**	0.91 ± 0.10	0.91 ± 0.11
L3	0.39 ± 0.06	0.74 ± 0.09	0.74 ± 0.09
L4	0.41 ± 0.08	0.76 ± 0.06	0.76 ± 0.06
L5	0.44 ± 0.08	0.78 ± 0.06	0.78 ± 0.06

*Note:* Bold values indicates Clinically significant difference.

### 3.2 Group B: DB Versus Clinician

The Kruskal–Wallis comparison test showed a statistically significant difference between all the values: RMS, minimum, and maximum values, with a *p*‐value of < 0.05 (Table 2, Supplement S2).

Descriptive statistics in Table 4 show 3 values with clinically significant differences in this group (> 0.50 mm): maxillary second premolars (0.54 mm), maxillary first premolars (0.52 mm), and mandibular second premolars (0.51 mm).

**TABLE 4:** Descriptive statistics Direct Bonding versus Virtual Clinician.

Teeth	RMS	Max	Min (−)
U1	0.40 ± 0.03	0.72 ± 0.08	0.72 ± 0.09
U2	0.38 ± 0.06	0.74 ± 0.12	0.74 ± 0.12
U3	0.39 ± 0.05	0.73 ± 0.07	0.73 ± 0.07
U4	**0.52 ± 0.15**	0.83 ± 0.07	0.84 ± 0.07
U5	**0.54 ± 0.15**	0.84 ± 0.10	0.85 ± 0.10
L1	0.41 ± 0.07	0.79 ± 0.13	0.79 ± 0.13
L2	0.43 ± 0.09	0.81 ± 0.17	0.81 ± 0.17
L3	0.40 ± 0.06	0.74 ± 0.09	0.74 ± 0.09
L4	0.39 ± 0.04	0.75 ± 0.04	0.75 ± 0.04
L5	**0.51 ± 0.17**	0.82 ± 0.08	0.82 ± 0.08

*Note:* Bold values indicates Clinically significant difference.

There were more discrepancies in Group A (DB vs. AI) with 4 values over 0.50 mm, when compared to Group B (DB vs. Clin) which only had 3 values over that threshold. In Group A, the teeth with the highest discrepancy were the mandibular and maxillary lateral incisors, while in Group B, the teeth with the most discrepancy were the maxillary second and first premolars.

## 4 Discussion

To our best knowledge, this study is the first of its kind since in vivo conditions are much more complex and unpredictable than in vitro experiments, and will hopefully bring some more insight and knowledge to this emerging field of research.

For our study, it is important to make the distinction between statistically significant differences (*p* < 0.05) and clinically significant differences. Assessment of clinical acceptability was based on the ABO objective grading system for dental casts. The ABO established that deviations from proper alignment of ≤ 0.50 mm are clinically acceptable. We found more discrepancies in Group A (DB vs. AI) than in Group B (DB vs. Virtual Clinician). We demonstrated that virtual indirect bonding, whether planned by AI or a clinician, will differ from the DB technique. The factors that could contribute to some of the differences with DB (in vivo) are isolation issues, patient head position, source of light, stress, and eye fatigue, among others.

In the literature, studies have been made comparing direct versus indirect bonding techniques, but mostly in vitro, since in vivo conditions are more complex and unpredictable than in vitro experiments. Advances in technology and 3D superimposition software features have improved, measuring the accuracy of indirect bonding [7]. Most of the related studies using 3D superimposition software to analyse bracket positioning discrepancies also have linear and/or angular measurements assessments [13].

Fiorillo et al. [7] evaluated the accuracy of a computer‐aided design and manufacturing indirect bonding technique using a customised 3D‐printed transfer tray and a flash‐free adhesive system for orthodontic bonding. They analysed 86 brackets and 20 buccal tubes. Mandibular second molars showed the highest positioning errors, whereas maxillary incisors reported the lowest values. The overall bonding inaccuracy measurement was 0.35 mm, below the clinical acceptability limit of 0.50 mm. It was concluded that the accuracy of a 3D‐printed customised transfer tray was generally high, with greater positioning errors for posterior teeth. In our study, we compared the DB that happens during a normal day in a busy orthodontic office to the virtual indirect bonding and a new AI‐supported bonding method. The differences found between the DB and Clin could be the result of salivation, patient head position, fatigue, stress, and/or the use of a new technology for virtual bonding by the clinician. The differences found between the DB and Clin could be a result of salivation, patient head position, fatigue, stress, and/or a result of using a new technology for virtual bonding by the clinician. The differences between the DB and the AI found in the upper laterals and premolars could be a result of the anatomical variations in those teeth; perhaps the AI model needs a larger dataset with different anatomical variations to improve the bracket positioning. Panayi et al. [1] studied DB, DB assisted with loupes, and indirect bonding. They had 18 patients and a sample of 298 permanent teeth. Their study found values higher than 0.45 mm in the posterior teeth region. All measurements were found to be statistically significantly different. They had linear and angular measurements instead of RMS values [1]. Therefore, we could not do a direct comparison to our study, but they had a similar sample to our study, and more discrepancies were found in the posterior area, which is consistent with our findings (Group A U4&5's), Group B (U4, 5&L5).

In our study, we did not use a coordinate measuring system since our goal was to discover if there were in fact bracket positioning differences regardless of the spatial direction of the differences. Previous studies reported that digital superimpositions provided more reliable and accurate data when compared with linear measurements [7, 14]. We decided to use the RMS value as our measuring parameter because it allowed us to see the absolute numerical difference in the bracket positioning [6]. It has been a popular measurement in the literature since it is less influenced by offset errors of positive or negative values that are usually related to linear measurements [7].

Limitations include the single‐centre design, limited sample size, use of one AI software system, and potential unaccounted intra‐patient correlations. We recognise that our current analysis does not model intra‐patient clustering. Mixed‐effects models would better account for these dependencies in future studies. Future studies with larger, multi‐centre samples and mixed‐model analysis are warranted to better capture clinical variability.

## 5 Conclusions

- Statistically and clinically significant differences were found when comparing AI and virtual indirect to DB.
- With our data, we could infer that if we compare AI to virtual indirect bonding, we will not find any clinical significant differences since the differences between both methods fall below 0.25 mm.

## Author Contributions

T.E. contributed to writing, methodology, and visualisation. N.S. and J.P. provided supervision. J.C.‐M. contributed to methodology.

## Ethics Statement

This study was approved by the Institutional Review Board of a major academic institution.

## Conflicts of Interest

The authors declare no conflicts of interest.

## Supporting information


**Data S1:** Differences in bracket positions (mm) between direct bonding and AI‐assisted bonding techniques. The figure illustrates positional deviations in millimetres measured between the two methods across all evaluated teeth.

**Data S2:** Differences in bracket positions (mm) between direct bonding and digital indirect bonding techniques. The figure shows positional discrepancies in millimetres assessed between the two approaches for all included teeth.

## Data Availability

The data supporting the findings of this study are available from the corresponding author upon reasonable request.
