# Supplementary figures and images for: Comparison Between Direct, Virtual Aided by Clinician and Artificial Intelligence Bonding Techniques in Orthodontics
Source: Orthod Craniofac Res. 2025 Oct 13;28(Suppl 1):S25–9. doi: 10.1111/ocr.70032 (PMC12927125; doi:10.1111/ocr.70032)

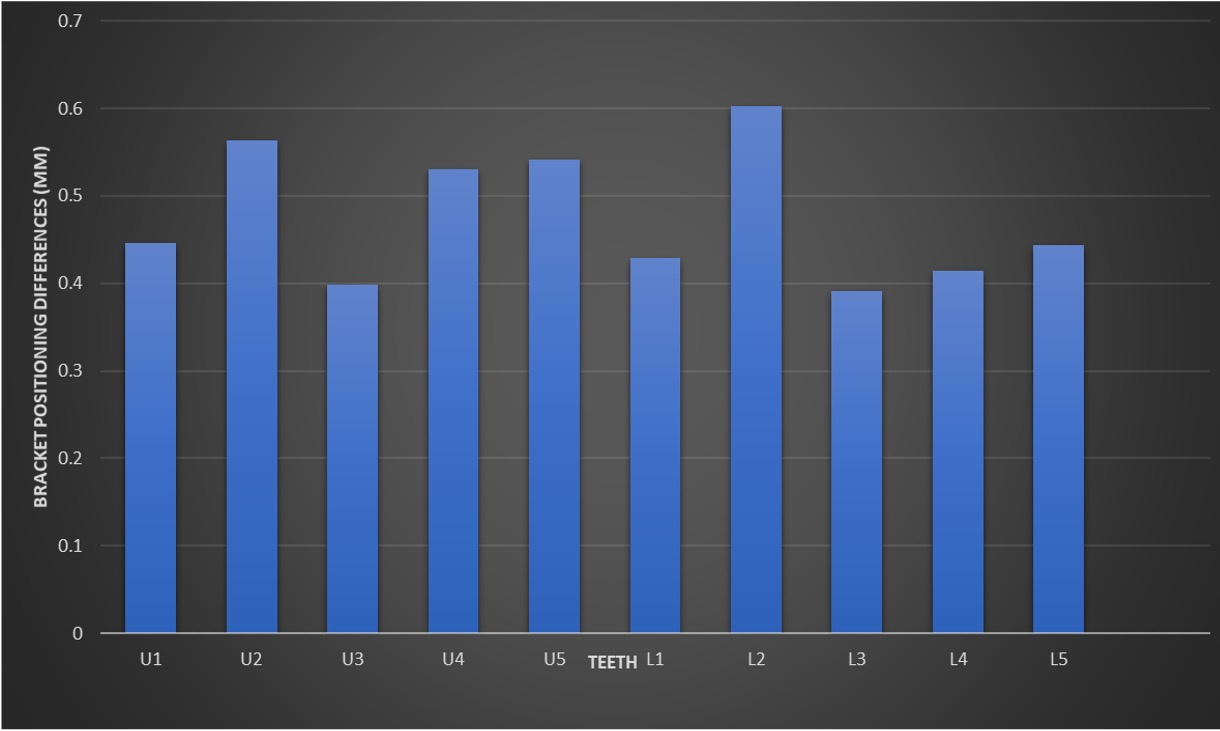

Supplement: Supplementary file 1 — Data S1: Differences in bracket positions (mm) between direct bonding and AI‐assisted bonding techniques. The figure illustrates positional deviations in millimetres measured between the two methods across all evaluated teeth. [file OCR-28-S25-s001.tiff]

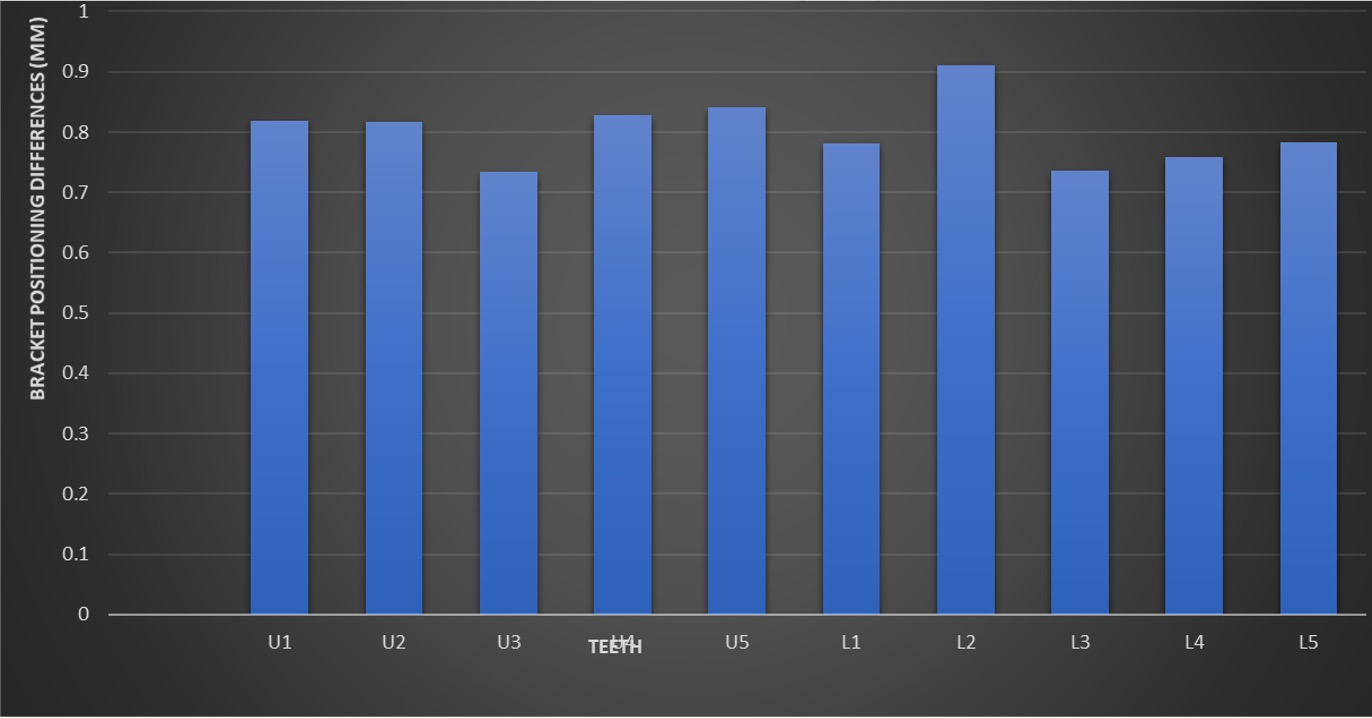

Supplement: Supplementary file 2 — Data S2: Differences in bracket positions (mm) between direct bonding and digital indirect bonding techniques. The figure shows positional discrepancies in millimetres assessed between the two approaches for all included teeth. [file OCR-28-S25-s002.tiff]
